# Severe Fever with Thrombocytopenia Syndrome Virus in Ticks Collected from Humans, South Korea, 2013

**DOI:** 10.3201/eid2008.131857

**Published:** 2014-08

**Authors:** Seok-Min Yun, Wook-Gyo Lee, Jungsang Ryou, Sung-Chan Yang, Sun-Whan Park, Jong Yeol Roh, Ye-Ji Lee, Chan Park, Myung Guk Han

**Affiliations:** Korea Centers for Disease Control and Prevention, Cheongwon-gun, South Korea

**Keywords:** severe fever with thrombocytopenia syndrome virus, SFTSV, severe fever with thrombocytopenia syndrome, SFTS, Phlebovirus, Bunyaviridae, humans, South Korea, viruses, vector, ticks, tickborne, tick-borne, Haemaphysalis longicornis, Amblyomma testudinarium, Ixodes nipponensis, Rhipicephalus microplus

## Abstract

We investigated the infection rate for severe fever with thrombocytopenia syndrome virus (SFTSV) among ticks collected from humans during May–October 2013 in South Korea*. Haemaphysalis longicornis* ticks have been considered the SFTSV vector. However, we detected the virus in *H. longicornis*, *Amblyomma testudinarium*, and *Ixodes nipponensis* ticks, indicating additional potential SFTSV vectors.

Severe fever with thrombocytopenia syndrome (SFTS) is an emerging disease characterized by fever and thrombocytopenia. The syndrome is caused by SFTS virus (SFTSV), a member of the family *Bunyaviridae*, genus *Phlebovirus* (*1*). SFTSV is related to, but distinctly different from, Heartland viruses, which were isolated in the United States (*2*).

The first case of SFTS was reported in China during 2010 (*1*), and in 2013, SFTSV infections were reported in South Korea and Japan (*3*–*5*). In South Korea, the first human case of SFTS was confirmed in May, 2013 (*3*). Although person-to-person transmission of SFTSV through contact with the blood or mucus of an infected person has been reported (*6*,*7*), the virus is primarily transmitted to humans by the bite of SFTSV-infected ticks. The virus has been detected in *Haemaphysalis longicornis* Neumann (bush tick) and *Rhipicephalus microplus* Canestrini (southern cattle tick) ticks (*1*,*8*).

*H. longicornis* ticks comprise the major population of ticks in the environment and have been considered the main vector for SFTSV (*9*,*10*). SFTSV has been detected in *H. longicornis* ticks collected from the environment by using the dragging or sweeping methods and from mammals. However, to our knowledge, the prevalence of SFTSV in ticks collected from humans has not been reported. To increase our understanding of SFTSV and its possible vectors, we determined the prevalence of SFTSV infection among various ticks collected from humans nationwide in South Korea during May–October 2013.

## The Study

We collected a total of 261 ticks (113 nymphal, 7 adult male, and 141 adult female ticks) from humans during May–October 2013. The ticks were placed in plastic tubes and transported to our laboratory for identification to species and developmental stage (*11*); we used a dissecting microscope for identification purposes. Tick samples were homogenized in 600 μL of phosphate-buffered saline (pH 7.0) containing 10% fetal bovine serum (GIBCO BRL, Grand Island, NY, USA), penicillin (500 IU/mL, GIBCO BRL) and streptomycin (500 μg/mL, GIBCO BRL) by using the Precellys 24 tissue homogenizer (Bertin Technologies, Bretonneux, France) and 2.8-mm stainless steel beads. We used a viral RNA extraction kit (iNtRON Biotechnology, Seongnam, South Korea) to extract RNA from the supernatant of the tick homogenates. To detect SFTSV RNA, we performed a 1-step reverse transcription PCR (RT-PCR) using a DiaStar 2× OneStep RT-PCR Pre-Mix Kit (SolGent, Daejeon, South Korea) with designed primers, MF3 (5′-GATGAGATGGTCCATGCTGATTCT-3′) and MR2 (5′-CTCATGGGGTGGAATGTCCTCAC-3′), under the following conditions: an initial step of 30 min at 50°C for reverse transcription and 15 min at 95°C for denaturation, followed by 35 cycles of 20 s at 95°C, 40 s at 58°C, and 30 s at 72°C, and a final extension step of 5 min at 72°C.

Of the 261 identified ticks, 4 nymphal ticks and 18 adult ticks had fed just before collection. The most abundant tick was *H. longicorni*s (81.2%, 212/261), followed by *Amblyomma testudinarium* Koch (6.5%, 17/261); *Ixodes nipponensis* Kitaoka and Saito (5.7%, 15/261); *H. flava* Neumann (5.4%, 14/261); and *H. japonica* Nutt and Warburton, *Ix. persulcatus* Schulze, and *Ix. granulatus* Supino (0.4% each, 1/261) (Table).

**Table:** Ticks collected from humans in South Korea during a study of severe fever with thrombocytopenia syndrome virus, 2013*

Tick species, developmental stage	No. ticks	No. pools	No. SFTSV-positive pools	MIR, %
*Haemaphysalis longicornis*				
Larvae	0	0	0	0
Nymphs	85	34	1	1.2
Adults, sex				
M	5	5	0	0
F	122	109	11	9.0
Subtotal	212	148	12	5.7
*Haemaphysalis flava*				
Larvae	0	0	0	0
Nymphs	9	4	0	0
Adults, sex				
M	2	2	0	0
F	3	3	0	0
Subtotal	14	9	0	0
*Haemaphysalis japonica*				
Larvae	0	0	0	0
Nymphs	0	0	0	0
Adults, sex				
M	0	0	0	0
F	1	1	0	0
Subtotal	1	1	0	0
*Amblyomma testudinarium*				
Larvae	0	0	0	0
Nymphs	16	13	4	25.0
Adults, sex				
M	0	0	0	0
F	1	1	0	0
Subtotal	17	14	4	23.5
*Ixodes nipponensis*				
Larvae	0	0	0	0
Nymphs	3	3	2	66.7
Adults, sex				
M	0	0	0	0
F	12	12	0	0
Subtotal	15	15	2	13.3
*Ixodes persulcatus*				
Larvae	0	0	0	0
Nymphs, sex	0	0	0	0
Adults				
M	0	0	0	0
F	1	1	0	0
Subtotal	1	1	0	0
*Ixodes granulatus*				
Larvae	0	0	0	0
Nymphs	0	0	0	0
Adults, sex				
M	0	0	0	0
F	1	1	0	0
Subtotal	1	1	0	0
Total				
Larvae	0	0	0	0
Nymphs	113	54	7	6.2
Adults, sex				
M	7	7	0	0
F	141	128	11	7.8
Total	261	189	18	6.9

*Pools of nymphal and adult ticks contained 1–30 ticks/pool and 1–5 ticks/pool, respectively. MIR, SFTSV minimum infection rate per 100 ticks (no. positive pools/total no. ticks assayed); SFTSV, severe fever with thrombocytopenia syndrome virus.

We divided the 261 ticks into 189 pools to detect the medium (M) segment gene of SFTSV by RT-PCR: 18 SFTSV-positive pools were detected. The SFTSV minimum infection rate per 100 ticks (MIR) was 5.7% in *H. longicornis* (12 pools), 23.5% in *A. testudinarium* (4 pools), and 13.3% in *Ix. nipponensis* (2 pools) ticks. In fed ticks, the MIR was 18.2% (4 pools), and in unfed ticks, the MIR was 5.9% (14 pools). The mean prevalence of SFTSV in the ticks in the study was 6.9%.

We identified the sequences for the SFTSV-positive tick pools by using the TA cloning method and a TOPO TA Cloning Kit (Invitrogen, Carlsbad, CA, USA). Because of a cloning failure, the sequence of 1 positive tick pool was not determined. The sequences obtained from 17 SFTSV-positive tick pools were submitted to GenBank (accession nos. KF781489–513).

Sequences from the SFTSVs detected showed 92.3%–98.8% identity with the partial sequence of the M segment from 17 SFTSV strains from South Korea and from 17 other SFTSV strains from China and Japan. Using the neighbor-joining method, we constructed a phylogenetic tree based on the partial M segment sequences (560 bp) obtained in this study and from SFTSV sequences in GenBank. The 17 strains from South Korea were closely related to the SFTSV strains from humans and ticks in China and Japan (Figure).

**Figure:**
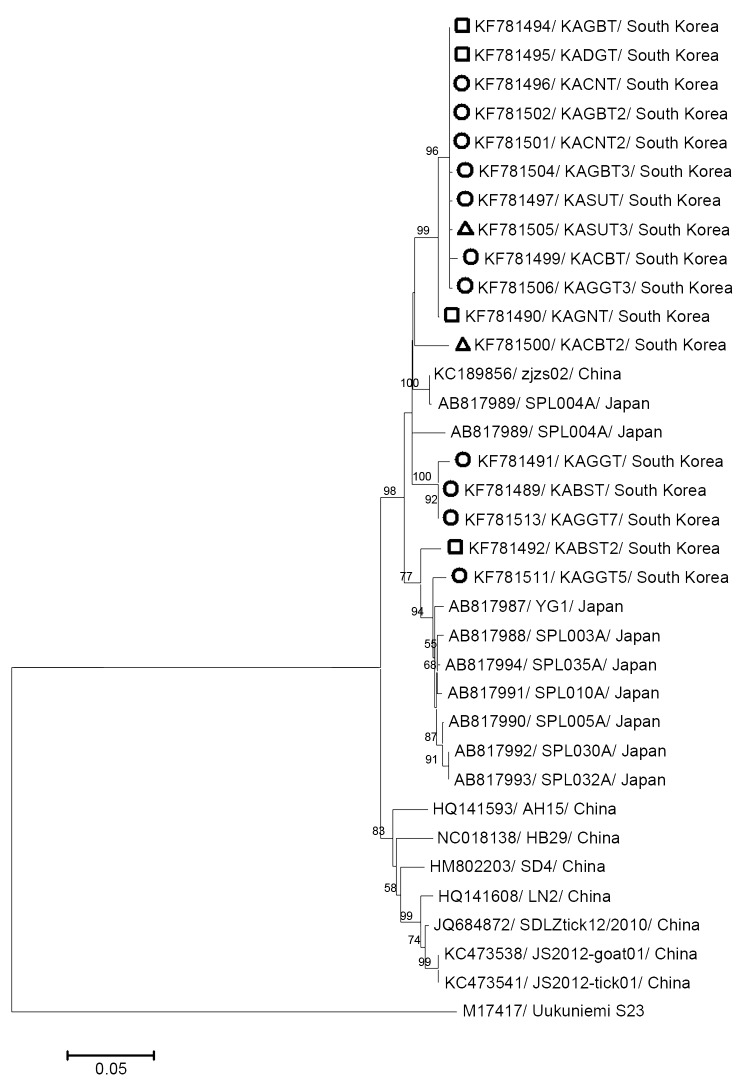
Phylogenetic analysis of severe fever with thrombocytopenia syndrome viruses based on the partial medium segment sequences (560 bp). The tree was constructed by using the neighbor-joining method based on the p-distance model in MEGA5 (*12*) (5,000 bootstrap replicates). Uukuniemi virus was used as the outgroup. Scale bar indicates the nucleotide substitutions per position. Among the 17 South Korean strains identified in this study, the Korean strains detected from *Haemaphysalis longicornis*, *Amblyomma testudinarium*, and *Ixodes nipponensis* ticks are marked with open circles, squares, and triangles, respectively. Numbers at nodes indicate bootstrap values.

## Conclusions

*H. longicornis* ticks have been considered the principal tick vector of SFTSV; there is limited information on SFTSV infection by other tick species. On the basis of our findings, we propose that *A. testudinarium* and *Ix. nipponensis* ticks, from which we detected SFTSV, might serve as potential vectors of this virus in South Korea. However, the presence of viral RNA in a tick does not confirm that the tick can transmit the virus. To our knowledge, *A. testudinarium* and *Ix. nipponensis* ticks have not previously been considered as SFTSV vectors.

Further studies, including studies to isolate SFTSV from infected tick vectors and laboratory vector competence studies, are needed to confirm whether *A. testudinarium* and *Ix. nipponensis* ticks transmit SFTSV to humans. We attempted to isolate SFTSV from the SFTSV-positive ticks in our study by using Vero E6 cells but were unable to do so.

Adults and nymphs of *Haemaphysalis* and *Ixodes* spp. have been collected most frequently from humans, but larvae have not been collected. We did not detect SFTSV from adult male ticks in our study; however, the number of collected ticks was small. In another study in South Korea, ticks were collected from medium- and large-sized mammals (*9*), and the species and developmental stage of ticks in that study were similar to those in our study. In that study, *H. longicornis* and *Ix. nipponensis* ticks were most frequently found on wild boar, water deer, roe deer, raccoon dog, Siberian weasel, Asian badger, and leopard cat. Although *A. testudinarium* ticks were not collected from mammals in that study, we did collected them from humans in our study.

The prevalence of SFTSV in *H. longicornis* ticks in our study was 5.7%. In 27 other localities in 9 South Korean provinces, the mean SFTSV MIR of *H. longicornis* ticks collected by dragging or sweeping in the environment was 0.5% (S.-W. Park et al., unpub. data). In a study of SFTSV in China, 0.7%–5.4% of the tick population was positive for SFTSV (*13*). In the United States, 0.02% of field-collected ticks were positive for Heartland virus (*14*). The SFTSV MIR for ticks in Japan has not been reported.

Farmers and agricultural and forest workers from rural areas are at high risk for SFTS because their work environment increases their risk of contact with SFTSV-infected ticks (*15*). In our study, most ticks were collected from persons who lived in rural areas. Although 18 persons were bitten by ticks infected with SFTSV, not all of them had signs or symptoms of SFTS. This finding suggests that further studies are needed to obtain a detailed understanding of SFTS as an emerging tickborne viral disease and to develop preventive measures for the disease.
